# Effect of Metal Oxides on the Pyrolytic Behavior and Combustion Performance of 5-Aminotetrazole/Sodium Periodate Gas Generators in Atmospheric Environment

**DOI:** 10.3390/ma18102249

**Published:** 2025-05-13

**Authors:** Chengkuan Shi, Zefeng Guo, Bohuai Zhou, Yichao Liu, Jun Huang, Hua Guan

**Affiliations:** School of Chemistry and Chemical Engineering, Nanjing University of Science and Technology, Nanjing 210094, China; chengkuan@njust.edu.cn (C.S.); guozf@njust.edu.cn (Z.G.); bohuai@njust.edu.cn (B.Z.); yichaoliu@njust.edu.cn (Y.L.); junhuang@njust.edu.cn (J.H.)

**Keywords:** 5-aminotetrazole, gas generator, metal oxide, thermal decomposition, combustion performance, thermal safety

## Abstract

5-aminotetrazole (5AT)-based gas generators, particularly the 5AT/NaIO_4_ system, have garnered interest for their high gas production and energy potential. This study investigates the impact of various metal oxides (MnO_2_, Al_2_O_3_, TiO_2_, CuO, Fe_2_O_3_, MgO, ZnO, and MoO_3_) on the thermal decomposition and combustion performance of 5AT/NaIO_4_. The REAL calculation program was used to infer reaction products, which indicated that the gas products are almost all harmless, with negligibly low percentages of NO and CO. Thermogravimetric analysis revealed that metal oxides, especially MoO_3_, significantly advance the decomposition process above 400 °C, reducing the activation energy by 130 kJ/mol and lowering critical ignition and thermal explosion temperatures. Combustion performance tests and closed bomb tests confirmed MoO_3_’s positive effect, accelerating reaction rates and enhancing decomposition efficiency. The system’s high Gibbs free energy indicates non-spontaneous reactions. These findings provide valuable insights for designing environmentally friendly gas generators, highlighting MoO_3_’s potential as an effective catalyst.

## 1. Introduction

5-aminotetrazole(5AT)-based gas generators have been widely used in rocket engines and solid propellants in recent years [1,2]. Due to the high nitrogen content of 82.3%, the potential gas-producing function of 5AT has attracted extensive attention in applications such as solid propulsion and airbags [3,4,5].

In order to improve the gas production capacity and gas production rate of 5AT and fully exploit its advantages of high nitrogen content, metal oxides are usually added to promote its decomposition process. Cao et al. [6] studied the reaction mechanism during the decomposition of 5AT by adding Al_2_O_3_ and MgO to 5AT/Sr(NO_3_)_2_ gas producing agent. Results showed that these two additives impacted the decomposition of 5-AT at a temperature above 310 °C, mainly focusing on the formation and decomposition of melamine, melem, and melon-like condensation products. Meanwhile, these two additives could also accelerate the decomposition of melon-like thermal stable polymers at a temperature above 590 °C. Zhou et al. [7] added CuO to 5AT/Sr(NO_3_)_2_ gas-producing agent. It is found that CuO mainly acts in the third stage of 5AT pyrolysis, where the activation energy of 5AT is significantly reduced, and the reaction mechanism changes from chemical reaction (F1) to diffusion (D3). Chen et al. [2] added MnO_2_ to 5AT, and with the presence of MnO_2_, the activation energy was reduced by 30.7 kJ/mol. Zhao et al. [8] investigated the effect of BiO_2_/PbO/TiO_2_/CoO on the decomposition process of 5AT/Sr(NO_3_)_2_ propellant. Experimental results indicated that Bi_2_O_3_ and PbO predominantly facilitated the thermal decomposition process of 5AT, while TiO_2_ and CoO primarily catalyzed the thermal decomposition of Sr(NO_3_)_2_. Zhang et al. [9] investigated the effect of Fe_2_O_3_/CuO/NiO on the decomposition process of 5AT, which revealed that the catalytic effect of TMOs on 5AT primarily performs on the polyaddition reaction of N-containing heterocycle and the ring opening reaction of melem. The major catalytic effect of TMO nano-particles is caused by the interaction action with dissociation products during high temperature ranges, subsequently contributing to catalyze combustion behaviors for 5AT. For the combustion process of 5AT, Cao et al. [10] studied the propellant samples with nano-ZnO and CuO. The influences of these two catalysts on the decomposition behavior are reflected in the flame structure. The addition of nano-CuO can make the flame diffusion model change and slightly increase the flame temperature, while nano-ZnO can lead to a small, pale flame with a dark zone that cannot be observed.

Based on the above research, it can be seen that the catalytic effect of metal oxides on oxidants and combustible agent formulations greatly reduces the overall activation energy of chemical formulations and increases the reaction rate of substances. Due to its tiny size, metal oxides can participate in the heat transfer process as a front-end “hot point”, which can regulate the combustion rate of gas generators and speed up the reaction process [11]. Most of the studies on gas-producing agents using 5AT as raw material focus on the kinetic analysis of the decomposition process of 5AT, which plays a role in laying the theoretical foundation of gas-producing agents. However, the research on the integral thermal decomposition and combustion performance of gas producer formulations remains to be explored. Particularly, for the comparison of the catalytic effects of metal oxides in the basic formula of the same system, only a few kinds are selected in the research process for research, and the catalytic effects of different metal oxides need to be compared in a unified manner. In the overall formulation, there is no strong or weak comparison of which metal oxide has a better effect on the increase in combustion rate and decomposition and combustion process. Previous studies on 5AT-based gas generators have primarily focused on kinetic analysis of decomposition but lack comprehensive investigations into integrated thermal decomposition and combustion performance.

In this study, 5AT/NaIO_4_ was selected as the basic gas generator. 5AT has a high nitrogen content, and its gas production capacity is well expected [12]. The oxidizer NaIO_4_ itself can also produce a large amount of gas, and the product is clean and environmentally friendly, which is a good oxidizer in the gas generator formulation [13,14]. In this study, eight commonly used metal oxides, MnO_2_, Al_2_O_3_, TiO_2_, CuO, Fe_2_O_3_, MgO, ZnO, and MoO_3_, were selected as additives, and the thermal decomposition behavior of the samples was studied by thermogravimetric analysis. The catalytic effects of eight metal oxides in the 5AT/NaIO_4_ system were compared. At the same time, the combustion performance characterization and the closed bomb device test were carried out, and the metal oxide with the best catalytic effect on the combustion behavior was determined to be MoO_3_ (reducing activation energy by 130 kJ/mol). The kinetic parameters of 5AT/NaIO_4_/MoO_3_ and the control group were calculated. This study has a guiding effect on the formulation of gas-producing agents in propellants and inflatable life-saving equipment such as automobile airbags. The results of this study show the effects of different metal oxides on the thermal decomposition behavior and combustion performance of different metal oxides as additives and provide valuable guidance for the formulation design and combustion simulation of the 5AT/NaIO_4_-based system.

## 2. Materials and Methods

### 2.1. Material Preparation

The information of the materials applied in the experiment is recorded in Table 1. The mass composition of the gas-producing agent powder mixture prepared in the experiment was 5AT/NaIO_4_/MOx = 40/60/5. The raw material 5AT was first ball-milled with ethanol as a solvent in a horizontal planetary ball mill at a speed of 200 r/min for 12 h due to its large particle size and then dried in an oven at 80 °C for 4 h. The obtained powder was passed through a 180-mesh sieve to screen the diameter of 60–80 μm for the preparation of the sample mixtures. The preparation process of the mixture was the same as that of 5AT. The various substances were weighed and mixed according to the formula ratio. Alcohol, as a lubricant, was poured into the ball mill, and the ball mill was mixed for 1 h. The mixed substances were taken out, dried, and screened, and the sieved particles with 120 mesh were subjected to the thermal analysis and combustion performance experiments.

In this study, eight kinds of 5AT gas generators containing different metal oxides as additives were designed. The ratio of oxidants is the same for all formulations. The proportions of the formulations are recorded in Table 2.

### 2.2. REAL Program Calculation Method

The parameters of the gas products produced by the combustion of pyrotechnic components in an ideal state were calculated using the REAL calculation program. The REAL program (Version 3.0) is a standard thermodynamic software. The chemical equilibrium products, composition, and state functions in a thermodynamic system, including the amount of gas and the maximum combustion temperature, can be calculated by programming. The catalytic ability of the reactions was ranked according to the calculated results of the addition of different metal oxides and verified by the experimental results of the combustion phenomenon.

In this paper, the gas production V and the adiabatic combustion temperature T and the combustion products are theoretically calculated by the Gibbs free energy minimization method. The calculation method is based on the following thermodynamic assumptions: the enthalpy of the reaction of the reactants is completely transferred to the reaction products; the temperature of all the gases involved is the same; and the exterior of the entire reaction is considered as an adiabatic model.

Based on the above assumptions, the enthalpy relationship between all reactants and combustion products at a specific pressure can be deduced by the following expression:

(1) $$\sum_{i=1}^{m}H_{reactants}=\sum_{i=1}^{n}H_{products}\;$$

where *m* and *n* are the quantities of reactants and combustion products, respectively.

For a combustion process at a specific pressure, the enthalpy change of each substance can be calculated by the following equation:

(2) $$H_{products}=\sum_{i=1}^{n}\int_{298}^{T}nC_{\left(p,i\right)}\;$$

where *C*(*p*,*i*) is a temperature-dependent function. For a reaction system containing combustion products of both gas phase and condensed-phase, the total Gibbs free energy of the reaction system can be expressed by the following equation.

(3) $$G=\sum_{i=1}^{p}n_{i}^{g}\left({\left(\frac{\mu^{0(g)}}{RT}\right)}_{i}+lnp+ln\frac{n_{i}^{g}}{n^{g}}\right)+\sum_{j=1}^{q}n_{j}^{c}\left(\frac{\mu^{0(c)}}{RT}\right)\;$$

where *p* and *q* represent the total number of species of substances in the gas and solid phases, respectively. ni and nj represent the molar quantities of substances *i* and *j*, respectively. *g* and c represent the substances in the gas and solid phases, respectively. μ0 represents the chemical potential at a specific pressure (1 atm). *r* is the standard gas constant. This equation is then calculated using the Lagrange multiplier method to obtain the equilibrium composition at the specified temperature and pressure.

The specific volume was calculated in standard state (101.3 kPa, 273.15 K) [15]. The calculation formula of the specific volume is as in the following equation.

V = 22.4 × ∑ni(g)(4)

where V is the specific volume (L·kg^−1^); ni(g) is the amount of substance of the i-th gaseous products of 1 kg gas generator.

### 2.3. Thermal Analysis Experiments

The thermal behavior of the samples was recorded using thermal analysis equipment. The TG curves of the samples were measured under air atmosphere using an HCT-2 microcomputer differential thermal balance (Beijing Hengjiu Scientific Instrument Factory, Beijing, China). The mass of the test samples was about 3 mg, and the TG-DTG-DSC curves of the samples were recorded at a heating rate of 10 °C/min from room temperature to 800 °C. In order to minimize experimental errors, each sample was tested three times under the same conditions, and each sample showed the same thermogravimetric regularity in parallel tests.

### 2.4. Characterization of Combustion Performance

**Figure 1:**
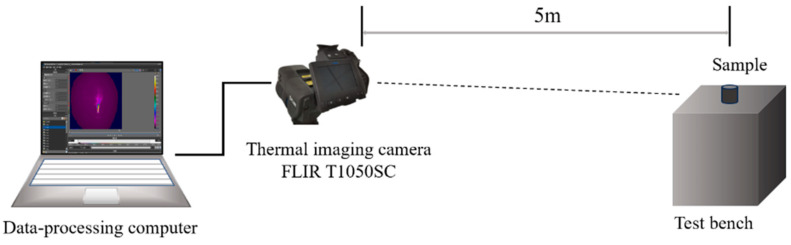
Sample test diagram.

The test sample was fixed on the test bench. The test distance was adjusted to 5 m, and the equipment was adjusted so that the lens was directed towards the flame when the sample was burning. A FLIR T1050SC thermal imaging camera, FLIR Systems, Wilsonville, OR, USA was used to record the combustion properties, such as the combustion temperature, the flame area, and the flame appearance. The obtained data was used to analyze the effects of different metal oxides as additives on the combustion process. Sample parameters: the density (ρ) is 1.7 g/cm^3^, the column diameter (Φ) is 18 mm, and the column height (H) is 15 mm. Thermal imager parameters: 8–14 μm, temperature range 0–2000 °C, frame rate 30 Hz. The test environment was atmospheric environment, the ambient temperature was 20 °C, and the pressure was standard atmospheric pressure. Figure 1 is a schematic diagram of the sample test. During the test, an electric heating wire was used to ignite the ignition powder on the surface of the sample, thereby igniting the gas-producing agents. The time from the ignition of the column until the flame is extinguished is recorded as the sample combustion time t/s. The combustion rate of different samples is calculated from the ratio of the height of the column sample to the combustion time, v = H/t. (in mm/s). The combustion temperature and flame area were calculated by ResearchIR software (Version 4.40.9.30). All samples were subjected to three sets of combustion tests, and the average combustion temperature and flame height showed consistent stability.

### 2.5. Closed Bomb Vessel Experiment

**Figure 2:**
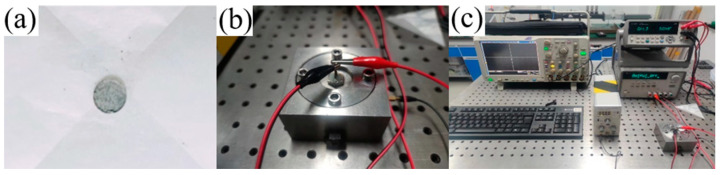
(**a**) Test sample. (**b**) Closed bomb vessel. (**c**) Closed bomb test system.

The closed bomb vessel test system is composed of a closed bomb vessel, a piezoelectric pressure sensor, a charge amplifier, a data collector, an analysis system, and a data processing system. The test sample is shown in Figure 2a. The closed bomb vessel is shown in Figure 2b, and the test system is shown in Figure 2c.

Pressure and time data for the ignition of 5AT/NaIO_4_/MOx mixtures were obtained using a 12 mL closed container for closed bomb testing. In the experiment, 80 mg of samples were weighed. The test samples were pressed with a mold of Φ8 mm size to obtain a flat disc sample with a size of 8 mm, a height of about 1.5 mm and a density of about 1.3 g/cm^3^. The test samples were ignited by applying a continuous electric current with the help of a nichrome wire igniter in a closed bomb containing a mixture of 5AT/NaIO_4_/MOx with different metal oxides, and the pressure vs. time data were recorded by a data acquisition system with the help of a pressure transducer. This experiment was used to determine the ability of the prepared samples of gas-producing agents to have practical application in airbags.

## 3. Results and Discussion

### 3.1. Calculation Results of REAL Program

In this study, the specific volume, gas production, and combustion temperature of eight metal oxides as additives for gas generators were calculated. The catalytic reaction ability of eight metal oxides was theoretically evaluated. The results of the calculations for the different formulations are listed in Table 3.

**Figure 3:**
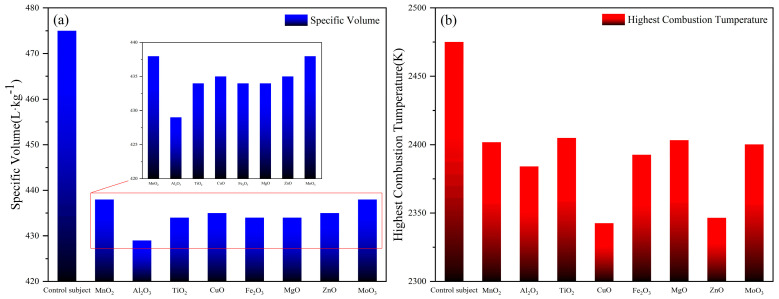
Comparison of parameters for 1#−8# samples. (**a**) Specific volume. (**b**) Highest combustion temperature.

The combustion performance parameters of eight gas generators based on 5AT/NaIO_4_ were calculated by the REAL program (above table). Figure 3a is the distribution of gas production of 1#-8# formula. The order of gas production for formulations 1#-8# is 8#/1# > 4#/7# > 3#/5#/6# > 2#. Since the base system is the same, the difference in gas production is not significant. However, the addition of metal oxides decreased the specific volume of the formulations by about 0.4 m^3^/kg, from 4.75 m^3^/kg to 4.35 m^3^/kg. A higher specific volume typically implies greater gas yield, which is desirable for applications requiring rapid pressure buildup (e.g., airbag deployment, solid rocket motors). Reduced specific volume can have a complex impact on the efficiency of gas generators. A lower specific volume may reduce gas production, but formulations with low specific volume typically have a higher energy density and more complete combustion, resulting in improved thermal efficiency despite the smaller volume of the gas. If the specific volume decreases, but the pressure increases, the system can achieve a higher work output per unit mass, thus offsetting the effect of lower gas production. Therefore, the actual inflation capacity of different formulations is to be evaluated in the closed bomb device test. The high gas output indicates that the reaction products can release more gas during the reaction. In practical application, whether the gas-producing agent has sufficient aeration capacity is actually considered in the comprehensive relationship between gas production volume and gas production rate. Therefore, we designed a closed bomb device experiment in the follow-up study. Figure 3b shows the distribution of combustion temperatures of the formulations 1#-8#. The combustion temperature order of the 1#-8# formulas is 3# > 6# > 1# > 8# > 5# > 2# > 7# > 4#. The calculated adiabatic combustion temperature is the maximum value of the theoretical combustion temperature, and the temperature will be relatively low in the actual combustion process. It is worth noting that in the 1#-8# formula with metal oxides, the maximum temperature of adiabatic combustion is 75–130 K lower than that of the control group at 2475 K. In all formulations, their high standard molar enthalpy of formation results in a calculated theoretical heat of combustion that is greater than that of the base formulation due to the addition of metal oxides as catalysts. The heat of combustion in descending order is 2# > 6# > 3# > 1# > 8# > 5# > 7# > 4#.

The main combustion products of the 5AT gas generator are N_2_, H_2_O, and CO_2_. The requirements for gas generators are that there are few solid particles in the gas, the gas is clean, and the gas temperature is low. The gas should be less ablative, corrosive, and toxic, and the combustion is less affected by the ambient temperature [15,16]. In the results of the REAL program calculations, it can be found that the gas products are almost all harmless, with negligibly low percentages of NO and CO. It is noted that a very small amount (<0.3%) of NO is contained, which is in accordance with the requirements for the gas products. In addition, in order to make the combustion of the gas generator more effective, a certain degree of negative oxygen system design is adopted, which will lead to the formation of CO. The control group had the highest CO content (2.16%) in the theoretical calculation, while the CO content of the sample with metal oxides decreased slightly (<1.65%), indicating that the metal oxides had a certain oxidation property and slightly weaker oxidizing ability. In fact, the theoretical calculation did not take into account the effect of oxygen in the atmospheric environment on combustion during the combustion process, and the actual combustion process would have relatively less CO, which is in line with the requirement of the product of the gas generator.

### 3.2. Thermal Decomposition and Reaction Mechanism of Gas Generators

Thermal analysis can be used to study the reaction and exothermic process between the components of the agent [17]. It is possible to determine when the decomposition process occurs and how it changes. In order to clarify the effect of metal oxides as catalytic additives on the reaction process of substances, the thermal decomposition process of 5AT-MOx was first analyzed by adding metal oxides with 5% mass content to the pure substance 5AT, respectively.

**Figure 4:**
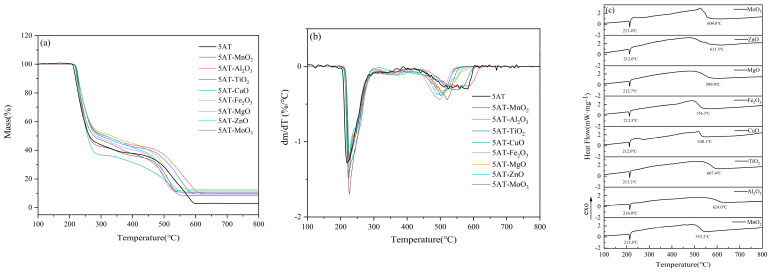
Thermal analysis curves of 5AT−MOx samples. (**a**) TG curves. (**b**) DTG curves. (**c**) DSC curves.

The DTG curves of the pure substance 5AT and eight 5AT-MOx samples with 5% metal oxides at 100–800 °C are shown in Figure 4a. The DTG curve shows that the mass decomposition of the sample is mainly composed of two parts. In the first pyrolytic weight loss stage, all thermal decomposition reactions start at 200 °C and end before 310 °C with a weight loss of about 58%. The second main pyrolytic weight loss stage starts at 400 °C and ends before 630 °C. Between 310–400 °C, there is a slow process of weight loss in thermal decomposition, with a mass loss of about 10%. For the 5AT-MOx sample with eight different metal oxides added, respectively, the temperature at the beginning and end of the DTG curve was nearly the same at the first major decomposition stage. The addition of eight metal oxides made the initial reaction temperature of the samples and the peak temperature of DTG curves slightly lag behind that of the pure 5AT but did not affect the overall trend of the first weight loss stage, indicating that the temperature range of the first major thermal decomposition was not significantly affected by the additives. In the second major decomposition stage, the catalytic effect of metal oxides is significantly reflected. Compared with the wide temperature range of the gradual decomposition of the pure substance 5AT between 400–630 °C, the temperature of the 5AT-MOx samples with the addition of Fe_2_O_3_, ZnO, MnO_2_, MgO, MoO_3_ and CuO to complete this decomposition stage is about 50 °C earlier, and the peak temperature of the DTG curve is 50–100 °C earlier, while Al_2_O_3_ and TiO have no significant effect on the third step of 5AT decomposition. Above 400 °C, the decomposition products of 5AT gradually polymerize on the surface of the liquid phase to form melamine, and continue to polymerize to form melon, etc., which gradually decompose with the increase of temperature to form DTG decomposition peaks over a wide temperature range [6]. Combined with the change of DTG curve, it can be inferred that the addition of metal oxides, as catalysts, attenuates the process of melamine polymerization, promotes the decomposition of melamine, makes the decomposition reaction more rapid and complete, and leads to the advance of the peak temperature and the termination temperature of the reaction.

In addition, on the DTG curve, only one obvious thermal decomposition peak appeared in each major decomposition stage of the 8 5AT-MOx samples, and the DSC curve showed the same thermal trend in Figure 4b. The temperature of the peak reached in each stage was slightly different due to the addition of metal oxides, indicating that the addition of catalyst did not significantly change the decomposition mechanism of the pyrolysis stage in the range of 200–310 °C and 400–630 °C. As can be seen from the DTG curves, all eight 5AT-MOx samples exhibit a similar thermogravimetric trend, which may be due to the similarity of the chemical bonds and molecular structures of the 5AT samples, and the fact that their chemical properties do not change with the addition of catalysts [10].

**Figure 5:**
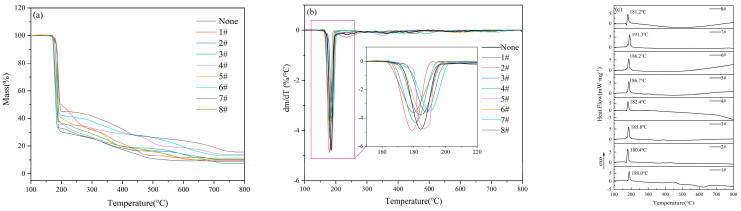
Thermal analysis curves of 5AT/NaIO_4_/MOx samples. (**a**) TG curves. (**b**) DTG curves. (**c**) DSC curves.

Unlike the multi-step decomposition process of pure 5AT and 5AT-MOx samples, the 5AT/NaIO4-MOx mixtures designed in this experiment are almost completely decomposed in a single-step reaction. The DTG curves of the eight mixture samples are shown in Figure 5a. The thermal decomposition of gas generators can be divided into two weightless stages. The first weight loss phase occurs before 200 °C, and the mass loss is about 65%. This phase is accompanied by exothermic effect, with the exothermic peak reaching a maximum between 181–189 °C. The second stage, which occurs between 300 and 500 °C, is a slow and continuous decomposition process that results in a weight loss of about 20%. Subsequently, melting of NaI occurs above 650 °C with a gradual decrease in mass. Over the course of the reaction, the total weight loss is approximately 90%, and the residual mass is approximately 10%.

The DSC curves of the eight mixture samples are shown in Figure 5b. It can be seen that there is a prominent exothermic peak in all the decomposition processes, indicating that the samples can release heat rapidly during the initial decomposition stage. It is noted that the DSC curve has a non-obvious recovery stage of exothermic peak, showing a step form of exothermic peak, which originated from the transformation of the 5AT decomposition product from the gas phase to the liquid phase. The liquid phase interface provides the medium for the reaction, and a series of decomposition, polymerization, and redox reactions take place on the surface of the liquid film, realizing the decomposition and reaction process of the substance from the solid phase to the gas phase and releasing heat [18].

The DSC curve showed that the peak temperature of the 5AT/NaIO_4_ sample was 187.2 °C with an exothermic quantity of 159.4 J/g. After the addition of different metal oxides, the exothermic quantities of the first stage reaction were 62.5 J/g (MnO_2_), 67.7 J/g (Al_2_O_3_), 72.1 J/g (TiO_2_), 59.6 J/g (CuO), 59.9 J/g (Fe_2_O_3_), 60.8 J/g (MgO), 63.8 J/g (ZnO), and 49.6 J/g (MoO_3_). The heat release from large to small is TiO_2_, Al_2_O_3_, ZnO, MnO_2_, MgO, Fe_2_O_3_, CuO, and MoO_3_.

Observing the decomposition process curves of the mixtures, the thermogravimetric loss of the mixtures mainly occurred in the temperature range of 160–200 °C. The initial transformation temperature Te, the peak temperature Tp of the DTG curve, and the peak temperature Tm of the DSC curve were counted in the first stage of the reaction, and the results are shown in Table 4. It is reasonable for the peak temperature of the exothermic peak to lag slightly behind the peak temperature of the DTG curve. The sample releases a large amount of heat and then reaches the peak of the DSC curve through the data acquisition of thermocouples, which makes the temperature of DSC have a certain lag. The three groups of data showed the same change trend and were sorted according to the order of initial transformation temperature, peak temperature of DTG curve, and peak temperature of DSC curve, which were Al_2_O_3_, MoO_3_, CuO, TiO_2_, MgO, Fe_2_O_3_, MnO_2_, and ZnO. The three sets of data have the same change trend, which also reflects the consistency of the test results. This can illustrate the strength of the catalytic effect of metal oxides on the 5AT/NaIO_4_ system.

The addition of metal oxides, for 5AT individuals, acted as a catalytic in the second major weight loss stage that promotes decomposition, promoting the cleavage of the polymer melamine, and making the reaction termination temperature advanced. The addition of metal oxides also has a considerable effect on gas generators. Although most of the mass is lost in one-step decomposition, the addition of metal oxides advances the peak temperature of this decomposition process by about 10 °C. The addition of metal oxides improved the heat transfer efficiency of the base system samples, leading to the advance of the reaction temperature. Due to the same decomposition trend, it can be inferred that metal oxides as catalysts improve the decomposition efficiency of gas generators without changing the decomposition process and decomposition mechanism.

The three sets of data showed the same trend, indicating that the addition of metal oxides did not change the original decomposition process of the system but only played a catalytic role in the local reaction process, resulting in the change in transformation temperature. It is worth noting that although the initial transformation temperature of all samples was earlier than that of the control group, the peak temperature of the DTG curves and the DSC curves were different. Among them, the peak temperature Tp of the DTG curves of the samples with the addition of MnO_2_, Fe_2_O_3_, MgO, and ZnO were lagging behind that of the control group, which indicated that the conversion rate of the reaction of the system was reduced after the addition of these four metal oxides. The peak temperature Tm of the DSC curves of the samples with the addition of MnO_2_ and ZnO lagged behind that of the control group, indicating that the catalytic effect of these metal oxides on the samples in the reaction process was not satisfactory. Comparatively speaking, the metal oxides with better catalytic effect are Al_2_O_3_, MoO_3,_ and CuO.

### 3.3. Combustion Performance of Gas Generators

**Figure 6:**
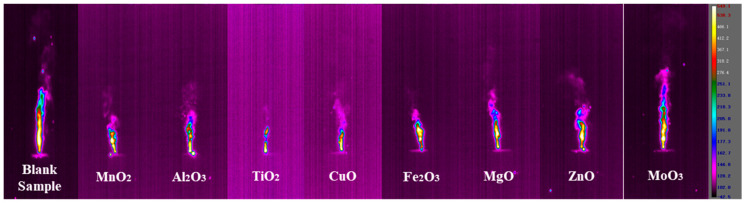
Screenshot of thermal image of the samples under stable combustion state.

The combustion behavior was recorded by the thermal imaging camera to get the combustion state of the samples during combustion. Figure 6 shows the screenshot of the thermal image of the sample when it reached a stable combustion state. The combustion flame height and flame area of the control group without metal oxide were 20.27 cm and 28.53 cm^2^, respectively. The flame area and flame height during the combustion process of the samples can reflect the intensity of the reaction to a certain extent [17]. The addition of metal oxides as catalysts can change the combustion state of the samples under the same conditions of the base formulation. From the thermal image screenshot of the combustion state, it can be found that the addition of metal oxides has weakened the flame height and area of the agent during combustion to a certain extent. Among them, the combustion state of the sample with the addition of MoO_3_ showed the best performance.

**Figure 7:**
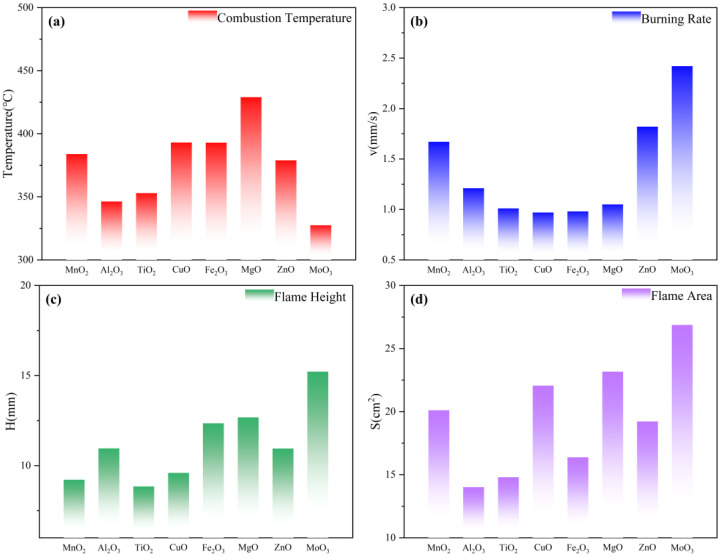
Performance parameters of samples in stable combustion state. (**a**) Combustion temperature. (**b**) Burning rate. (**c**) Flame height. (**d**) Flame area.

The combustion temperature and combustion rate can evaluate the performance of the gas generators. In general, it is required that the temperature of the gas generator is as low as possible and that it reacts rapidly and produces a large amount of gas [15]. The temperature and combustion velocity of the gas generator samples with different metal oxides as additives in the stable combustion state are shown in Figure 7a,b. The combustion temperature of the control group was 381.5 °C, and the combustion velocity was 4.1 mm/s. The combustion velocities of the gas generators with the addition of metal oxides somewhat showed the opposite of the combustion temperature. Among them, the combustion temperature of the formula with MnO_2_, CuO, Fe_2_O_3_, and MgO increased, while the combustion temperature of the formula with Al_2_O_3_, TiO_2_, ZnO, and MoO_3_ decreased. All the formulations showed a tendency to decrease the combustion rate, indicating that the addition of metal oxides inhibits the combustion rate of gas generators. CuO has the most obvious inhibitory effect on the combustion rate. MoO_3_ performs the best among the selected metal oxides due to its lower combustion temperature and relatively higher combustion rate, which is fully in line with the formulation design requirements of gas generators. MoO_3_ has better combustion performance as an additive, which is evidenced by the highest flame height and the largest flame area in Figure 7c,d. From the perspective of combustion performance, the combustion temperature from high to low is 6# > 4# > 5# > 1# > 7# > 3# > 2# > 8#. The burning speed from high to low is 8# > 7# > 1# > 2# > 6# > 3# > 5# > 4#. Evaluating the addition of eight metal oxides in terms of combustion performance, MoO_3_ was the most effective.

Although the trace addition of metal oxides can achieve the catalytic effect on the decomposition of gas generators on the microscopic scale, in the actual combustion process, because it cannot form a macroscopic heating surface of the thermal diffusion rate, it will absorb a part of the heat of the reaction of the agent during the heat transfer process, resulting in the reduction of the burning rate [11]. However, once the metal oxide additive is involved in the reaction, it will increase the combustion temperature of the reaction to a certain extent, which is reflected in the actual testing process. The addition of metal oxides caused the reaction region of 5AT/NaIO_4_/MOx to concentrate on the surface of the agent rather than the meteorological combustion area after decomposition, leading to the reduction in the combustion flame height and the decrease in the flame area. Different metal oxides have different properties of their own, which also leads to differences in combustion phenomena. It was noted that the diffusion of hot solid particles from the solid phase to the meteorological zone was clearly observed during the combustion process for the formulation with the addition of MoO_3_. This behavior directly leads to the alleviation of the metal oxides on the combustion temperature accumulation effect in the solid phase region. At the same time, the high-temperature diffusion particles provide an environment for the combustion of decomposition products, resulting in a better combustion performance of the formulation 5AT/NaIO_4_/MoO_3_, which is manifested in the higher flame height and larger flame area. It can be speculated that on the combustion surface, MoO_3_ is embedded in the form of particles. When 5AT decomposes, these particles are expelled into the gas phase region by the gas products, accelerating the backward movement of the combustion surface. The burning state of the agents can also be confirmed in the thermal image, and the more particles in the combustion flame, the faster the sample burns. During the combustion of 5AT/NaIO_4_/MoO_3_, there is a certain amount of glowing solid particles released from the solid phase into the gaseous region. While metal oxides inhibit flame propagation, MoO₃ strikes the best balance—lowering temperature without severely compromising combustion efficiency. Its ability to enhance gas-phase reactions makes it ideal for clean, high-output gas generators, though particle emissions demand careful engineering. Other oxides (e.g., CuO) are better suited for high-temperature niches.

### 3.4. Closed Bomb Device Test of Gas Generators

**Figure 8:**
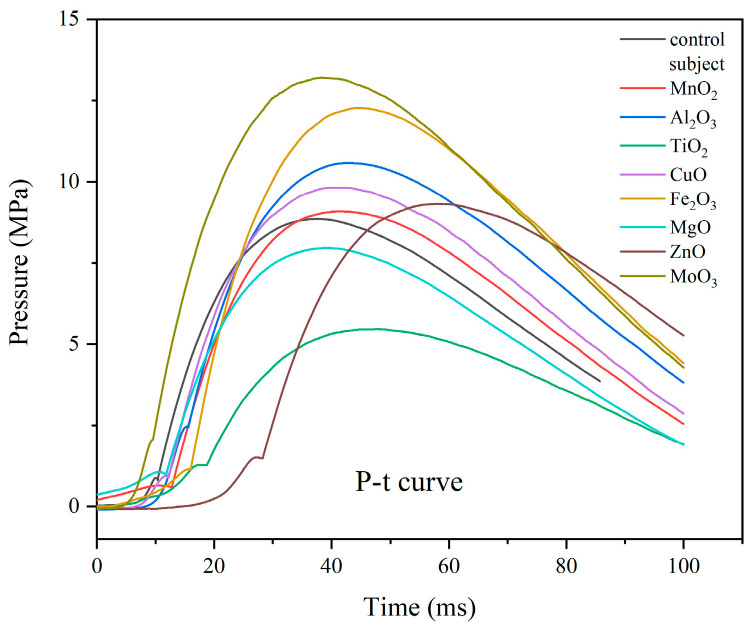
P-t curve of the samples.

The pressure inside a closed bomb device is an important indicator of gas production [15]. The pressure-time curves obtained by the closed bomb device test during the combustion of eight 5AT/NaIO_4_/MOx mixtures are shown in Figure 8. The maximum pressure (P_max_) and the time taken to reach the maximum pressure (t_max_) were obtained from the P-t curves and recorded in Table 5. The average rate of pressure rise from the start of combustion to the maximum pressure is ΔP_max_/Δt_max_, which reflects the rate of chemical reaction. As can be seen from Figure 8, the time of the samples with added metal oxides to reach the peak pressure was slightly longer than that of the control group. The peak pressure of 3# and 6# samples with TiO_2_ and MgO added, respectively, is smaller than that of the control group. The peak pressure of 3# formula with TiO_2_ added has the most obvious change in the peak pressure, with a maximum pressure of 5.60MPa. The addition of the rest of the metal oxides makes the pressure peak increase, among which the best performance is the 8# sample with MoO_3_ added, which not only has the highest peak pressure but also has the shortest time to reach the peak pressure. Compared with the ΔP_max_/Δt_max_ data (ΔP_max_/Δt_max_ = 0.241 MPa/ms in the control group), the gas production performance of the 8# formula is better. The ΔP_max_/Δt_max_ value of the 8# sample is the highest, indicating that the formula 5AT/NaIO_4_/MoO_3_ has the best gas production performance. The value of the 5# formula is lower than the 8# formula but better than other formulas. The ΔP_max_/Δt_max_ values of the 2# and 4# samples were slightly higher than that of the control group. The ΔP_max_/Δt_max_ values of the 1#, 3#, 6#, and 7# samples were all smaller than that of the control group, and although the peak pressures of the 1# and 7# samples were higher than that of the control group, the ratios of the 1# and 7# samples were smaller than that of the control group due to the longer time taken to reach the peak pressure. In addition, the 7# formula has the longest time to reach the peak pressure. In terms of combustion temperature, the gas producer used in the airbag should have a low combustion temperature to ensure that the output gas can be quickly cooled down to meet the needs of the application. The 8# sample with MoO_3_ added still had the highest combustion pressure despite the lowest combustion temperature, indicating its maximum gas production capacity. The addition of MoO_3_ to the 5AT/NaIO_4_ gas producing agent system can increase the gas production rate and gas production while maintaining the lowest combustion temperature, which is conducive to the practical application of the system as an airbag. At the same time, it is necessary to pay attention to the calculation of gas production and design a reasonable gas producing agent content to ensure safe and reliable inflation behavior.

### 3.5. Studies of Thermodynamic Parameters and Thermal Safety

The objective of studying kinetics is to obtain a kinetic model and to calculate the kinetic triple factors *A*, *Eα*, and *f*(α), where *f*(α) is the differential form of the kinetic reaction mechanism function, which represents the functional relationship between reaction rate and reaction conversion. The integral form of the reaction mechanism function is usually denoted as *g*(α) [19].

According to the Arrhenius equation [20], the kinetic equation under non-isothermal conditions can be expressed as Equation (5):

(5) $$\beta \frac{d\alpha }{dT}=Af\left(\alpha \right)exp(-\frac{Ea}{RT})$$

where *A* is the pre-exponential factor, *Eα* is the activation energy, *R* is the molar gas constant (8.314 Jmol^−1^K^−1^), *T* is the thermodynamic temperature, *α* is the reaction conversion rate, and *β* is the linear heating rate. In this study, the pyrolysis kinetics of samples will be analyzed using model-free methods. The conversion rate α can be defined as Equation (6) [21], and the mass values are derived by the TG curves:

(6) $$\alpha =(m_{0}-m_{t})/(m_{0}-m_{f})$$

where *m*_0_ is the initial mass; *m*_t_ is the mass at a given temperature; and *m*_f_ is the final mass.

The advantage of the model-free method (Flynn-Wall-Ozawa method) is that it can bypass the choice of the reaction mechanism function and directly find the reaction activation energy *Eα*, avoiding the possible errors due to the assumption of the reaction mechanism function.

The Flynn-Wall-Ozawa (FWO) method is an integral method with an algebraic expression as shown in Equation (7), which can be used to make a plot of lg*β* versus 1/*T* from the peak temperature data or temperature data obtained at a certain conversion and fit it linearly. Based on a slope of 0.4567*E*/*RT*, the activation energy, *Eα*, is calculated [22,23].

(7) $$lg\beta =lg\frac{AE_{a}}{Rg(\alpha )}-2.315-0.4567\frac{E_{a}}{RT}$$

**Figure 9:**
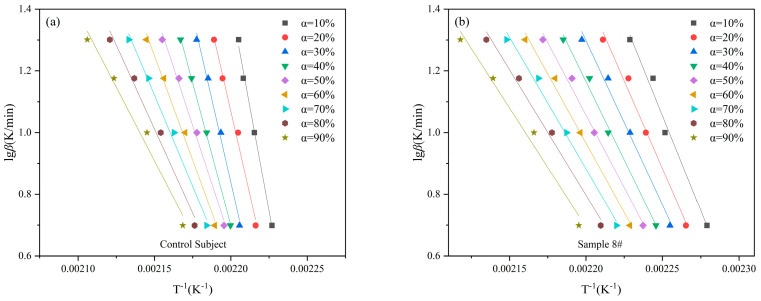
Fitted curves of decomposition kinetics by FWO method. (**a**) Control subject. (**b**) Sample 8#.

As can be seen from the previous discussion, the 8# sample with MoO_3_ has a lower decomposition temperature and better combustion performance, which indicates that the 8# sample is a more potential gas producing agent. In this study, the activation energy (*E*α) and pre-exponential factor (*A*) of the control subject and 8# sample were calculated using the Flynn-Wall-Ozawa (FWO) method. The data were plotted with 1/*T*_p_ as the x-axis and lg*β* as the y-axis, and linear regression was performed to obtain the straight lines as shown in Figure 9a,b. The calculated kinetic parameters are presented in Table 6. The activation energy values obtained from both methods are nearly identical, and the correlation coefficients of the fitted curves are close to 0.99, indicating a good fit. The activation energy of the control subject is significantly higher than that of the 8# sample, suggesting that after the addition of MoO_3_, the decomposition reaction of the substance is more likely to occur. We noticed that the activation energy values calculated by the FWO method varied greatly with the change of conversion rate. Therefore, the Vyazovkin method was used for calculations at the same time.

For the Vyazovkin method [24], the Eα value at different conversions can be obtained by minimizing the following function:

(8) $$\Phi \left(E\alpha \right)=\sum_{i=1}^{n}\sum_{j\neq i}^{n}\frac{I(E\alpha ,T\alpha ,i)\beta j}{I(E\alpha ,T\alpha ,j)\beta i}$$

where *i*, *j* are the different heating rates, and *n* is the total number of heating rates.

Andrzej Mianowski [25] proposed a simple calculation of the Vyazovkin method. The activation energy Eα for a constant conversion degree is possible to directly determine from Equation (9).

Eα = RT_i_T_j_ [ln(q_j_/q_i_) + 2ln(T_i_/T_j_)]/(T_j_ − T_i_), α = const, j > i (9)

For N heating rate, the activation energy is calculated as the geometric mean according to Equation (10).

(10) $$E\alpha ={\left(detE\right)}_{N-1}^{1}$$

N = 4 means:

(11) $$E=\sqrt[{3}]{E_{12}E_{23}E_{34}}$$

The activation energy at each conversion rate calculated using this method and the average activation energy are recorded in Table 6. The average values calculated by the two methods are similar.

A low activation energy barrier means that the material requires less external energy to initiate decomposition or combustion. Mechanical stresses (e.g., friction, impact) are more likely to form “hot spots”, increasing the risk of accidental ignition. MoO₃ may act as a catalyst, reducing the thermal stability of 5AT and promoting rapid energy release upon impact. In the case of continuous vibration, the friction between the chemical particles may accumulate heat, which may cause thermal disasters. It is necessary to use encapsulation technology and thermal insulation layer coating technology to prevent accidental damage. At the same time, the storage environment is strictly monitored to avoid heat accumulation at ambient temperature and possible mechanical vibrations. The box is designed to protect against the impact of accidental drops.

Thermal safety and thermal kinetic parameters are crucial for high-nitrogen energy-containing materials. The activation energy and correlation coefficient of control subject and sample 8# obtained by the FWO method are listed in Table 6. Compared to the activation energy of the control subject, which is 302 kJ/mol, there is a roughly 130 kJ/mol decrease in the activation energy of sample 8#, indicating that the system is more reactive with the addition of metal oxide MoO_3_. Thermal kinetic data obtained by the FWO method are used as input parameters here. As the heating rate approaches zero (β → 0), the values of *T*_00_, *T*_e0_, and *T*_p0_ correspond to the values of *T*_0_, *T*_e_, and *T*_p_ [26], which are obtained by linear regression as follows:

*T*_0/e/p_ = *T*_00/e0/p0_ + *bβ* + *cβ*^2^+ *dβ*^3^ + *eβ*^4^ (12)

where *b*, *c*, *d*, and *e* are coefficients. *T*_e0_ equals self-accelerating decomposition temperature, *T*_SADT_, referring to the lowest ambient temperature at which temperature increase of a chemical substance is at least 6 °C in a specified commercial package during a period of seven days or less. During storage and handling process, *T*_SADT_ is crucial for accessing safety management of self-reactive propellants, pyrotechnics, and explosives.

Critical ignition temperature (*T*_TIT_) and thermal explosion temperature (*T*_b_) are both vitally pivotal parameters for energetic materials [26]. Therein, *T*_b_ is defined as the lowest temperature to which a specific charge might be heated without undergoing thermal runaway. *T*_TIT_ corresponds to the substitution of *E*_e0_ and *T*_e0_, while *T*_b_ is obtained by substituting *E*_p0_ and *T*_p0_.

(13) $$T_{TIT/b}=\frac{E_{0}-\sqrt{E_{0}^{2}-4E_{0}RT_{e0/p0}}}{2R}$$

As listed in Table 7, high values of *T*_TIT_ and *T*_b_ represent that the occurrence of transition from thermal degradation to thermal explosion is not easy to happen. However, this temperature is still low compared to 5AT.

Gibbs free energy(Δ*G*^≠^), enthalpy(Δ*H*^≠^), and entropy of activation (Δ*S*^≠^) are critical thermodynamic parameters of activation, which could be obtained by Equations (14)–(16) when the values determined at *T* = *T*_p0_, *E_α_* = *E*_k_, and *A* = *A*_k_ [27,28].

*A*exp(−*E*_α_/*RT*) = (*k*_B_*T*/*h*) exp(−Δ*G*^≠^/*RT*) (14)

Δ*H*^≠^ = *E*_α_ − *RT* (15)

Δ*G*^≠^ = Δ*H*^≠^ − *T*Δ*S*^≠^ (16)

where *k*_B_ is the Boltzmann constant, and *h* is the Plank constant. Computed values of Δ*G*^≠^ and Δ*H*^≠^ are all positive, indicating that the sample endothermic decomposition reaction could not proceed without heating, which belongs to the non-spontaneous reaction. It is noted that the temperature range here is less than 200 °C. Therefore, attention should be paid to the design of the thermal insulation layer during the application process to prevent possible safety hazards due to a temperature increase caused by the external environment.

The reaction rate (*k*) of thermal decomposition can be calculated by Formula (17).

log *k* = log *A* − *E*_α_/2.3*RT* (17)

The activation energy *E*α and pre-exponential factor *A* calculated by the FWO method were substituted into this formula, and the ambient temperature was set to 25 °C (298.15 K). In contrast, it can be seen that the reaction rate of 5AT/NaIO_4_ pyrolysis is accelerated after the addition of metal oxide MoO_3_.

## 4. Conclusions

In this paper, eight different metal oxides as additives are comprehensively evaluated for gas generator formulations. From the perspective of high gas production, low combustion temperature, and non-toxic gas products, the calculation of the REAL program shows that the combustion products of various formulations meet the requirements of gas generators. The results of thermal analysis showed that the metal oxides that had a good promoting effect on the decomposition of 5AT and the decomposition of 5AT/NaIO_4_ were Al_2_O_3_, MoO_3_, and CuO, among which MoO_3_ had the best effect. The combustion performance test showed that the addition of MoO_3_ resulted in a lower combustion temperature and a relatively faster burning rate of the gas generator, which was also confirmed in the closed bomb test. From the perspective of combustion performance, the 5AT/NaIO_4_/MoO_3_ sample has a higher flame height and a larger flame area, indicating that it has a larger gas production and is the most promising gas generator. Thermal and combustion analyses confirm that 5AT/NaIO₄/MoO₃ exhibits superior flame propagation and gas yield, offering practical insights for propellant and gas-generator design. It is worth noting that the critical ignition temperature (*T*_TIT_) and thermal explosion temperature (*T*_b_) were reduced compared with the control group after the addition of MoO_3_, and the temperatures of the two were similar, indicating that the thermal stability of the system was reduced. In the process of gas generator production and application, attention should be paid to the design of the insulation layer and the temperature monitoring of the storage environment.

## Figures and Tables

**Table 1:** Material related information.

Chemicals	Formula	Specification	Manufacturer
5-aminotetrazole	5AT	AR, Particle size 60–80 μm	Sinopharm Chemical Reagent Co., Ltd., Shanghai, China
Sodium Periodate	NaIO_4_	AR, Particle size 2–10 μm	Sinopharm Chemical Reagent Co., Ltd., Shanghai, China
Manganese dioxide	MnO_2_	Content ≥85.0%, Black poeder	Sinopharm Chemical Reagent Co., Ltd., Shanghai, China
Aluminum oxide	Al_2_O_3_	AR, Amorphous white powder	Sinopharm Chemical Reagent Co., Ltd., Shanghai, China
Titanium dioxide	TiO_2_	AR, Particle size 2–4 μm	Sinopharm Chemical Reagent Co., Ltd., Shanghai, China
Copper oxide	CuO	AR, Black powder	Sinopharm Chemical Reagent Co., Ltd., Shanghai, China
Ferric oxide	Fe_2_O_3_	AR, Red brown powder	Sinopharm Chemical Reagent Co., Ltd., Shanghai, China
Magnesium oxide	MgO	AR, White fine Powder	Sinopharm Chemical Reagent Co., Ltd., Shanghai, China
Zinc oxide	ZnO	AR, White powder	Sinopharm Chemical Reagent Co., Ltd., Shanghai, China
Molybdenum oxide	MoO_3_	AR, Cyan powder	Sinopharm Chemical Reagent Co., Ltd., Shanghai, China

**Table 2:** 5AT gas generator formulas with different metal oxide additives.

Number	5AT (Mass Content/%)	NaIO_4_ (Mass Content/%)	Metal Oxides
Control subject	40	60	——
1#	40	60	5% MnO_2_
2#	40	60	5% Al_2_O_3_
3#	40	60	5% TiO_2_
4#	40	60	5% CuO
5#	40	60	5% Fe_2_O_3_
6#	40	60	5% MgO
7#	40	60	5% ZnO
8#	40	60	5% MoO_3_

**Table 3:** Calculated results of the REAL program for gas generator samples.

	Control Subject	1# MnO_2_	2# Al_2_O_3_	3# TiO_2_	4# CuO	5# Fe_2_O_3_	6# MgO	7# ZnO	8# MoO_3_
H(kJ·mol^−1^)	−491.99	−791.93	−1313.88	−1078.43	−590.69	−749.39	−1238.20	−707.34	−757.71
S(kJ·mol^−1^·K^−1^)	6.68	6.46	6.42	6.42	6.45	6.43	6.44	6.44	6.43
T(K)	2475.04	2401.82	2384.23	2404.87	2342.69	2392.63	2403.22	2346.54	2400.23
V(m^3^·kg^−1^)	4.75	4.38	4.29	4.34	4.35	4.34	4.34	4.35	4.38
U(kJ·kg^−1^)	−967.35	−1229.78	−1742.82	−1512.66	−1025.36	−1183.08	−1672.03	−1142.75	−1195.95
CO_2_(44)	0.1275	0.1333	0.1297	0.1279	0.1381	0.1322	0.1281	0.1377	0.1283
CO(28)	0.0216	0.0130	0.0153	0.0164	0.0100	0.0138	0.0163	0.0102	0.0162
N_2_(28)	0.2556	0.2434	0.2438	0.2437	0.2436	0.2436	0.2437	0.2436	0.2437
NO(30)	0.0028	0.0027	0.0020	0.0021	0.0023	0.0024	0.0021	0.0023	0.0021
NO_2_(46)	0	0	0	0	0	0	0	0	0
H_2_O(18)	0.0902	0.0877	0.0878	0.0874	0.0886	0.0874	0.0874	0.0886	0.0873
MOx	-	0.0461	0.0476	0.0474	0.0367	0.0397	0.0473	0.0380	0.0283

**Table 4:** Three thermodynamic temperatures of samples obtained from thermal analysis test.

	*T*_e_	*T*_p_	*T*_m_
control subject	184.2	185.9	187.2
MnO_2_	183.9	187.9	189.0
Al_2_O_3_	176.7	178.7	180.4
TiO_2_	180.4	184.7	185.8
CuO	177.4	181.1	182.4
Fe_2_O_3_	182.1	186.2	186.7
MgO	181.0	186.0	186.2
ZnO	184.1	190.4	191.3
MoO_3_	176.9	179.4	181.2

**Table 5:** Closed bomb test results of samples.

Formulation	Time to Reach the Peak Pressure/Δt_max_ ms	Peak Pressure/ΔP_max_ MPa	ΔP_max_/Δt_max_ MPa/ms
control subject	37.24	8.96	0.241
1#	41.46	9.24	0.223
2#	43.52	10.80	0.248
3#	47.70	5.60	0.117
4#	40.44	10.08	0.249
5#	44.28	12.48	0.282
6#	38.52	8.16	0.212
7#	58.00	9.48	0.163
8#	38.14	13.44	0.352

**Table 6:** Kinetic parameters activation energy calculated by FWO method and Vyazovkin method.

α	Control Subject			Sample 8#		
	*E*a (kJ/mol)		R^2^ (FWO)	*E*a (kJ/mol)		R^2^ (FWO)
	FWO	Vyazovkin		FWO	Vyazovkin	
0.1	489	561	0.9952	224	235	0.9830
0.2	395	407	0.9919	207	202	0.9884
0.3	394	386	0.9932	194	185	0.9903
0.4	336	339	0.9992	185	184	0.9916
0.5	274	265	0.9932	171	1666	0.9922
0.6	246	239	0.9938	163	156	0.9932
0.7	215	208	0.9916	155	147	0.9919
0.8	198	188	0.9852	148	139	0.9899
0.9	174	166	0.9804	140	132	0.9815
average	302	307		176	172	

**Table 7:** Thermal safety and thermal kinetic parameters of control subject and sample 8# based on FWO method.

Parameters	Control Subject	Sample 8#
*E*a/(kJ·mol^−1^)	302	176
lg*A*/(s^−1^)	42.9	28.2
*T*_00_/K	435.4	432.9
*T*_SADT_/K	446.3	439.3
*T*_p0_/K	454.9	443.8
*T*_TIT_/K	451.9	448.8
*T*_b_/K	460.7	453.5
Δ*G^≠^*/(kJ·mol^−1^)	42	47
Δ*H^≠^*/(kJ·mol^−1^)	298	172
Δ*S^≠^*/(J·mol^−1^)	563	282
log *k*	−10.0	−2.7

## Data Availability

The original contributions presented in this study are included in the article. Further inquiries can be directed to the corresponding author.
